# Stability of vancomycin hydrochloride solutions in high concentration and extended time of infusion

**DOI:** 10.1186/2197-425X-3-S1-A717

**Published:** 2015-10-01

**Authors:** MA Peterlini, APS Barbosa, M Pedreira

**Affiliations:** Federal University of São Paulo, Pediatric Nursing, São Paulo, Brazil

## Introduction

Vancomycin hydrochloride is an antimicrobial commonly used and studied in the treatment of patient with healthcare-associated infections. However, there are few studies related to this drug stability during administration. In intensive care units, mainly pediatric and neonatal, the clinical conditions and specific needs of patients may require longer time of vancomycin hydrochloride infusion or concentrated solutions, different of literature recommendations of a final dilution of 2.5 to 5.0 mg/ml in one hour of infusion. There is a lack of evidences for nursing practice regarding vancomycin hydrochloride infusion in higher concentration and longer time.

## Objectives

To determine the stability of vancomycin hydrochloride solutions at a concentration of 10mg/mL, according to two infusion periods.

## Methods

Experimental study that simulated the nursing clinical practice of intravenous administration of vancomycin hydrochloride solutions at a concentration of 10mg/ml. The stability was determined by high-performance liquid chromatography (HPLC) and pH in two conditions of temperature, 22°C( ± 1) and 37°C( ± 1), and two exposure times, 60 and 120 minutes. The concentration and pH were monitored after drug reconstitution with water for injection, after dilution with sodium chloride 0.9% and after 60 and 120 minutes of infusion. The sample was collected in triplicate to pH analyzes, and each triplicate was analyzed by HPLC in quintuplicate to determine a final concentration. Data were analyzed by using the mean and standard deviation (m ± SD).

## Results

Of the 60 pH analyzed values of vancomycin solutions, no substantial variations were observed (Tables 1). HPLC analyzes resulted in 120 measured concentrations; at 22°C( ± 1) an increased concentration of 4.03% and 21.11% were identified, at 60 and 120 minutes, respectively (Table 1). Solutions exposed to 37°C( ± 1) resulted in an increased concentration of 4.59% in 60 minutes and a decreased concentration of 10.28% in 120 minutes (Table 1). It is important to highlight that medication preparation was accomplished in syringes and the measured initial concentrations were different than 10 mg/mL.

**Table 1:** Concentration, variation ratio and pH.

Experimental situations	Concentration (mg/mL)		Concentration variation	pH(unit)	
	Initial	Final	(%)	Initial	Final
22ºC( ± 1)60 minuts	9.17 ± 0.56	9.54 ± 1.32	4.03	3.59 ± 0.03	3.64 ± 0.02
22ºC( ± 1)120 minuts	8.43 ± 0.57	10.21 ± 1.41	21.11	3.63 ± 0.05	3.65 ± 0.01
37ºC( ± 1)60 minuts	9.57 ± 0.74	10.01 ± 0.45	4.59	3.62 ± 0.02	3.65 ± 0.01
37ºC( ± 1)120 minuts	10.71 ± 1.60	9.61 ± 1.33	10.28	3.61 ± 0.02	3.63 ± 0.02

## Conclusions

Vancomycin hydrochloride solutions diluted at 10 mg/ml presented a clinically relevant alteration when infused during 120 min, despite the analyzed temperature.

